# Computer-aided identification of protein targets of four polyphenols in Alzheimer’s disease (AD) and validation in a mouse AD model

**DOI:** 10.7555/JBR.32.20180021

**Published:** 2019

**Authors:** Chaoyun Li, Ping Meng, Benzheng Zhang, Hong Kang, Hanli Wen, Hermann Schluesener, Zhiwei Cao, Zhiyuan Zhang

**Affiliations:** 1. Institute of Pathology and Neuropathology, University of Tuebingen, Tuebingen D-72076, Germany; 2. Department of Pathology, Nanjing Medical University, Nanjing, Jiangsu 211166, China; 3. School of Life Sciences and Technology, Tongji University, Shanghai 200092, China.

**Keywords:** Alzheimer's disease, polyphenol, INVDOCK, cerebral amyloidosis, behavioral deficit

## Abstract

Natural polyphenols are a large class of phytochemicals with neuroprotective effects. Four polyphenolic compounds: hesperidin, icariin, dihydromyricetin and baicalin were selected to evaluate their effects on Alzheimer’s disease (AD). We analyzed by an inverse docking procedure (INVDOCK) the potential protein targets of these polyphenols within the KEGG AD pathway. Consequently, their therapeutic effects were evaluated and compared in a transgenic APP/PS1 mouse model of AD. These polyphenols were docked to several targets, including APP, BACE, PSEN, IDE, CASP, calpain and TNF-α, suggesting potential *in vivo* activities. Five month old transgenic mice were treated with these polyphenols. Icariin and hesperidin restored behavioral deficits and ameliorated Aβ deposits in both the cortex and hippocampus while baicalin and dihydromyricetin showed no substantial effects. Our findings suggest that hesperidin and icariin could be considered potential therapeutic candidates of human AD.

## Introduction

Alzheimer’s disease (AD) is now the most common form of dementia among the elderly population, accounting for more than half of cases in clinical series and at autopsy^[1]^. It is clinically characterized by progressive cognitive deterioration, neuropsychiatric and behavioral symptoms. AD pathogenesis is supposed to be triggered by the accumulation of amyloid beta (Aβ) in brain parenchyma^[2]^.

Natural polyphenols are a large class of phytochemical compounds that are composed of aromatic rings and one or more phenolic rings. They may interact with the aromatic residue present in amyloidogenic proteins and then inhibit the self-assembly process resulting into amyloid fibril formation^[3]^. Baicalin, icariin, dihydromyricetin (DHM) and hesperidin are drug components of Traditional Chinese Medicine (TCM). Baicalin is one of the main bioactive flavone glucuronides derived from *Scutellariabaicalensis Georgi*, which is one of the most popular traditional medical herbs in Asia^[4]^. Icariin is the most active compound of *Epimedium* species, which have been used for more than one thousand years to treat chronic nephritis, osteoporosis, asthma, cardiovascular problems and hepatitis in East Asia. *Hoveniadulcis*, the premier anti-hangover herbal medicine, was listed in *Tang Materia Medica*, China’s first pharmacopeia published in the year of 659. DHM, also known as Ampelopsin, is a natural supplement derived from *Hoveniadulcis*. Hesperidin is a naturally occurring bioflavonoid found abundantly in citrus fruits, like *Citrus sinensis* and *Citrus reticulate*^[5]^. All of these compounds have pleiotropic biological properties, including anti-oxidant, anti-inflammatory, anti-hypotensive, anti-microbial and anti-carcinogenic activities. Moreover, all of them exert little adverse effect, have low or no cytotoxicity, and could be able to cross the blood-brain barrier (BBB). They might have potential in the treatment of neuroinflammatory and neurodegenerative diseases.

Identification of AD-related proteins that directly interact with drugs is of great importance for understanding anti-AD activities. Therefore, we tried to predict potential target proteins of these four polyphenols by an inverse docking procedure (INVDOCK). INVDOCK has been employed to conduct computer-automated inverse-docking searches of every entries in the Protein Data Bank (PDB) database to identify potential protein targets of a small molecule^[6]^. It has been successfully used in identifying protein targets of bioactive ingredients for explaining the molecular mechanisms underlying their biological activities^[7]^. Further, the compounds were analyzed in a transgenic AD mouse model.

## Materials and methods

### Inverse docking

An inverse docking procedure, INVDOCK, has been introduced to select proteins which directly bind to these four polyphenols^[7]^. INVDOCK has a built-in biomolecular cavity database derived from PDB entries. For the purpose of saving computing time, a subset of cavity database including only human proteins presented in “Alzheimer’s disease pathway” from Kyoto Encyclopedia of Genes and Genomes (KEGG) pathway database^[8]^, rather than the whole cavity database resulting from all PDB entries, was employed to run INVDOCK. The energy score for each docked conformation (compound and cavity) was calculated, aiming to determine if one protein could be bound by the compound. Only those docked conformation with interaction energy below δE_threshold,_ which is derived from empirical calculation, was supposed as binding targets of this polyphenol compound^[6]^.

### Transgenic mice

Heterozygous male transgenic APP/PS1-(21) mice were obtained from Prof. M. Jucker^[9]^ and were bred with female wild-type C57BL/6J mice. All offspring were characterized by PCR genotyping and kept as previously described^[10]^. The experiments were approved by Regional Administrative Council of Tuebingen (HF2/11) and licensed according to The German Animal Welfare Act (TierSchG) of 2006.

### Materials

Baicalin, icariin, DHM and hesperidin (all>98%) were purchased from Huike Botanical Development Co., Ltd (Xi’an, China), MR Natural Product Co., Ltd. (Xi’an, China), Lyphar Biotech Co., Ltd (Xi’an, China) and Tokyo Chemistry Co., Ltd (Tokyo, Japan), respectively. All of them were suspended in 1% carboxymethylcellulose (CMC, Blanose®, Hercules-Aqualon, Düsseldorf, Germany) and administered orally in a dose of 100 mg/kg (12.5 mg/mL). Control mice received the same volume of CMC.

### Treatment

Because it was not possible to obtain so many mice at a time, seven groups of transgenic mice (*n*= six for each group) were utilized for three sequentially experimental treatments. All mice were five months old and treated by daily gavage for ten days. In experiment Ⅰ, baicalin group received 100 mg/kg of baicalin; in experiment Ⅱ, icariin group received 100 mg/kg of icariin and DHM group were treated by 100 mg/kg of DHM; in experiment Ⅲ, hesperidin group received 100 mg/kg of hesperidin. In each experiment, gender-, age- and bodyweight matched transgenic mice, as control (control Ⅰ, control Ⅱ and control Ⅲ group), received the same volume of 1% CMC dissolved in water.

### Immunohistochemistry

Polyphenol-treated and control mice were sacrificed by CO_2_ euthanasia after the ten day treatment. Brain tissues were processed and stained by immunohistochemistry as described previously^[9]^, with antibodies against β-amyloid (1:100; Abcam, Cambridge, UK) for Aβ deposits.

After immunostaining, tissue sections were examined by light microscopy (Nikon Cool scope, Nikon, Düsseldorf, Germany). Aβ plaques in cortex and hippocampus were counted by a certain diameter and clear deposition for plaques. Then, the percentages of areas with specific immune-reactivity (IR) in the cortex and hippocampus were analyzed using the software Meta Morph Offline 7.1 (Molecular Devices, Toronto, Canada).

### Nest-building assay

Nest-building assay was performed as reported previously^[11]^. It was used to determine the potential changes of affiliative social behavior of transgenic mice following treatment. The presence and quality of the nest construction was evaluated on a three-point scale by three independent observers blinded to treatment categories.

### Social interaction assay

The social interaction assay was performed according to previous studies^[12^–^13]^ with minor modifications. As a broad screen of activities, the resident-intruder assay was video-recorded to quantify the independent and interactive behaviors of control and polyphenol-treated mice as a resident in the absence (first 15 minutes) and presence (second 15 minutes) of an intruder mouse, combined with analysis of movement to evaluate overall activity level and overt neurobiological differences. By playing back these videotapes, behavioral events of resident mice were counted by three independent observers blinded to group assignment. The mouse movements were tracked and the positions recorded for each frame in the computing environment Java, using the ICY software^[14]^. Then, the total distance traveled per transgenic mouse could be easily calculated using the mouse behavior analysis software developed in our lab.

### Statistical analysis

Data are showed as medians and quartiles for continuous data. The differences of behavioral events, plaque counts or IR area percentages between baicalin and control groups, or hesperidin and control groups were analyzed by exact nonparametric Mann-Whitney *U* test. All of the parameters described above among icariin, DHM and their control groups, were compared using the Kruskal-Wallis test, followed by *post-hoc* tests. Statistical significance was defined as *P* values≤0.05. All statistical analyses were performed with the GraphPad Prism 5.0 Software (GraphPad Software Inc., San Diego, CA, USA).

## Results

### Candidate target proteins of baicalin, icariin, DHM and hesperidin

**Fig.1:**
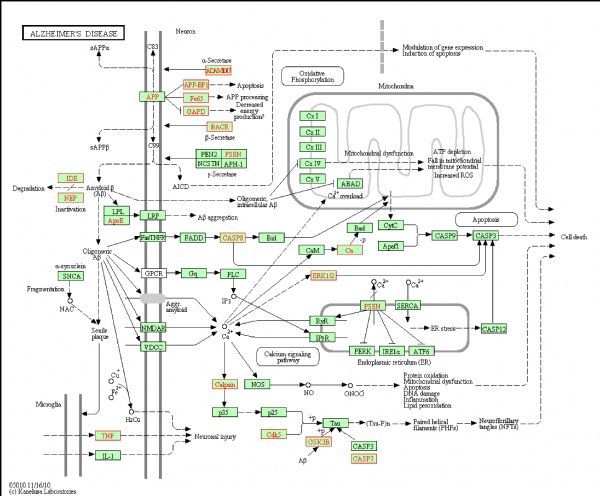
Distribution of target proteins of baicalin in the “Alzheimer’s disease pathway”.

**Fig.2:**
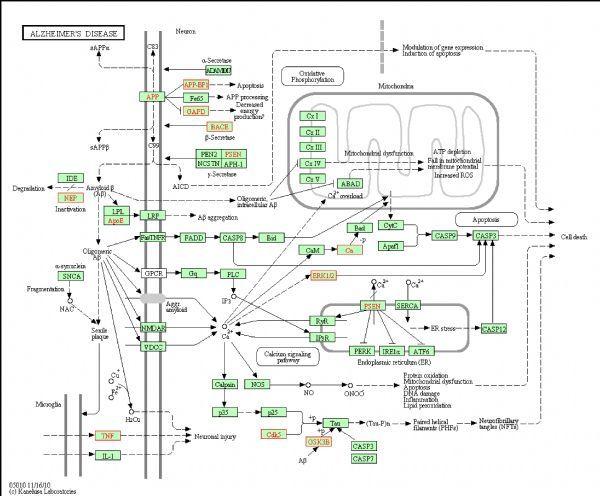
Distribution of target proteins of icariin within the “Alzheimer’s disease pathway”.

**Fig.3:**
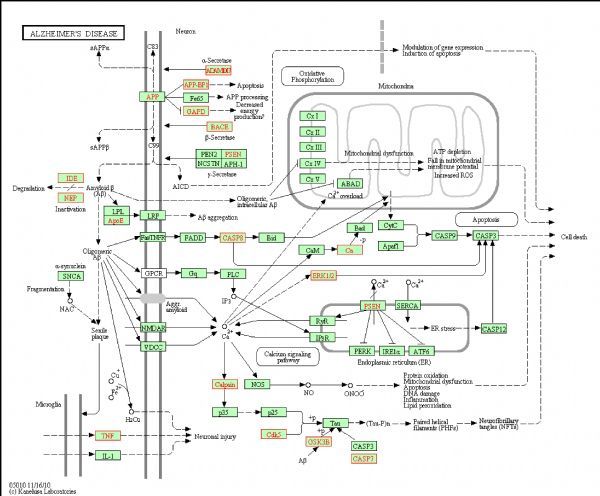
Distribution of target proteins of dihydromyricetin within the “Alzheimer’s disease pathway”.

**Fig.4:**
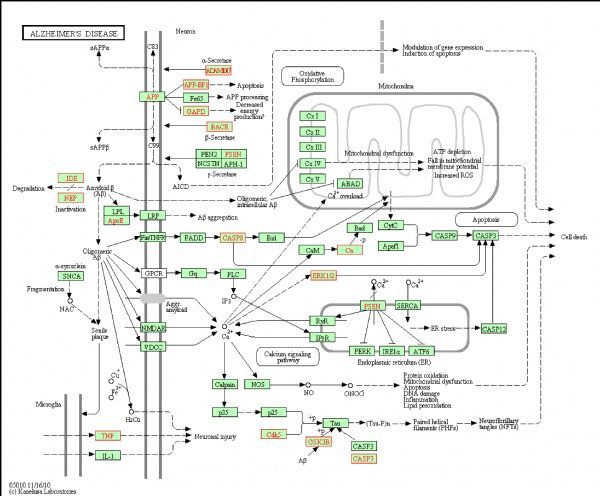
Distribution of target proteins of hesperidin in the “Alzheimer’s disease pathway”.

**Fig.5:**
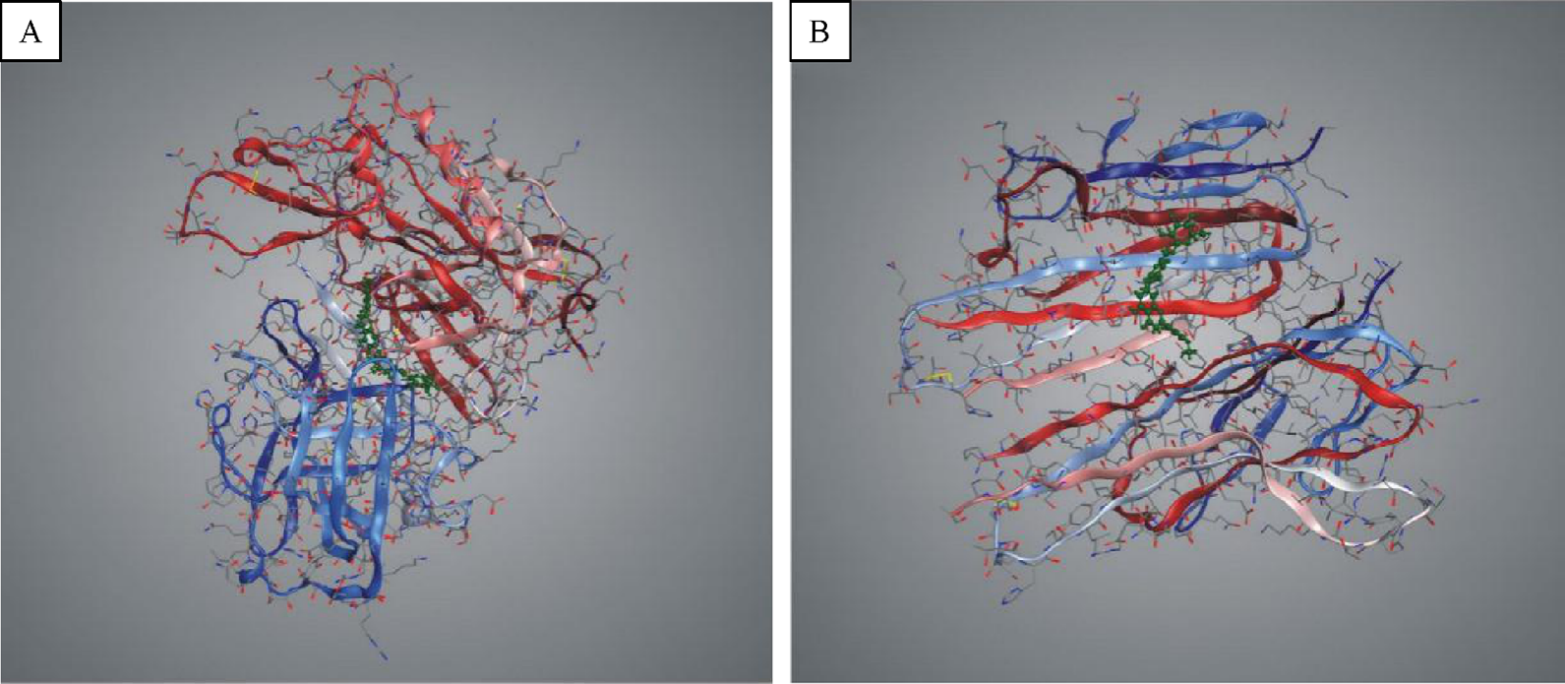
Illustration of hesperidin molecule docked in BACE1 and TNF-α by INVDOCK program.

For selecting potential targets of baicalin, DHM, icariin and hesperidin, INVDOCK was adopted to analyze proteins of the “Alzheimer’s disease pathway” from Kyoto Encyclopedia of Genes and Genomes (KEGG) database^[8^,^15]^. Finally, 18 (baicalin), 12 (icariin), 17 (DHM) and 16 (hesperidin) proteins were predicted as potential AD-related targets by virtual docking (***Fig. 1***–***4***, all targets highlighted by red boxes). As illustrations, models of hesperidin binding to BACE1 and TNF-α are shown in ***Fig. 5***.

### Effects of four polyphenols on Aβ accumulation in brains of APP/PS1 mice

The data presented above suggest that all four polyphenols, with only minor differences, have similar binding patterns and might have similar anti-AD effects by modulating amyloidosis. To make an *in vivo* comparison of drug effects, we used a transgenic APP/PS1 mouse model, which is a widely applied animal model of cerebral amyloidosis. We evaluated the potential therapeutic effects of the four polyphenols on Aβ deposition and behavioral dysfunction.

**Fig.6:**
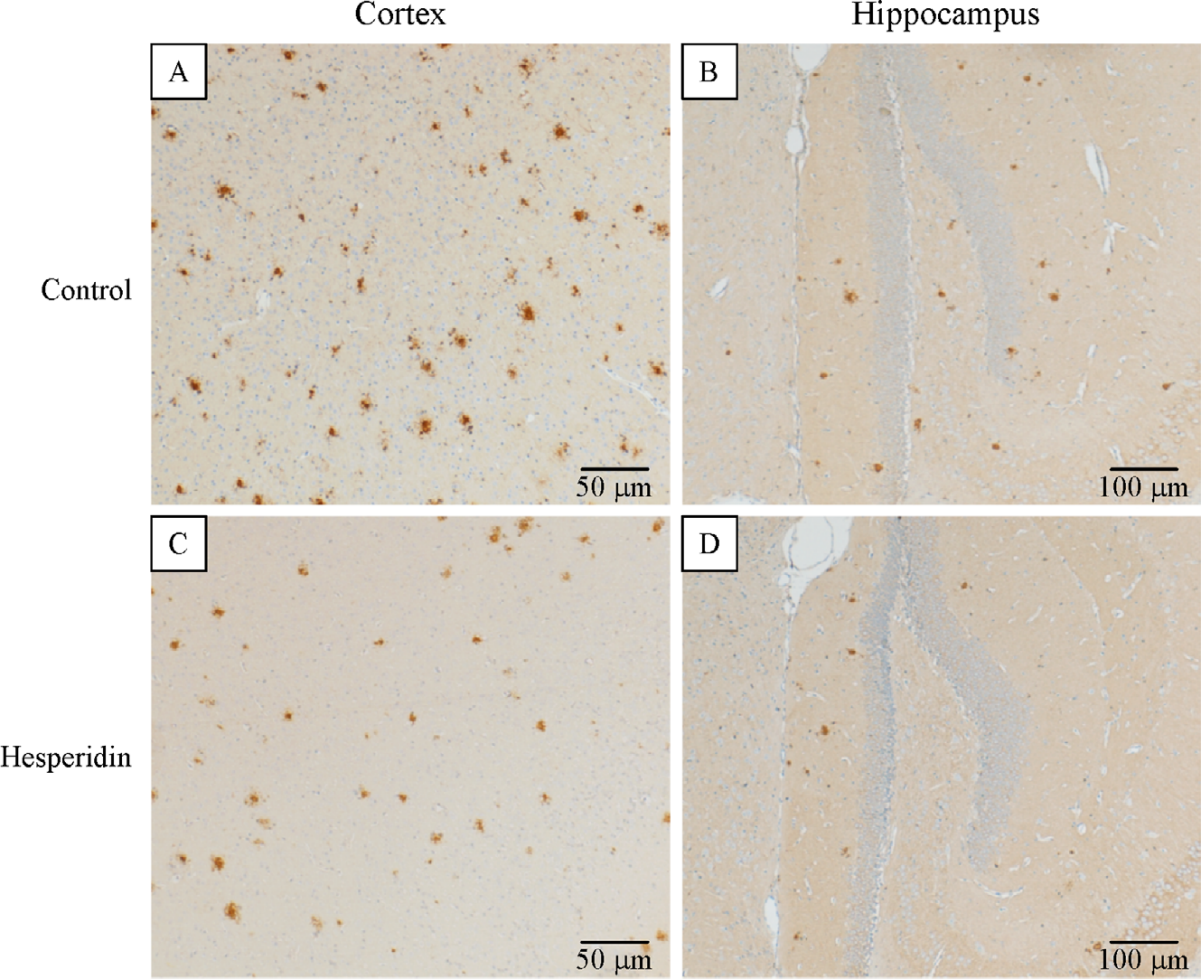
**Hesperidin reduced cerebral amyloidosis in transgenic APP/PS1 mice. **

In the five month old transgenic APP/PS1 mice, amyloid plaques were distributed throughout the cortex, some of them were small with dense core plaques and some were larger plaques with a dense core including a large halo of diffuse amyloid (***Fig. 6A***,*** C***). In the hippocampus, plaque density was distinctly lower (***Fig. 6B***, ***D***).

**Fig.7:**
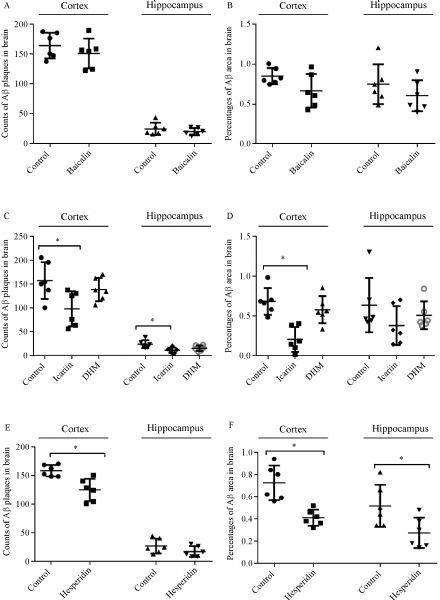
**Effect of four polyphenols on cerebral amyloidosis of transgenic APP/PS1 mice. **

Polyphenols were given daily to mice by gavage for a period of ten days. The mice were then killed and the effect on amyloid plaque was quantified by immunostaining for β-amyloid. In the cortex of baicalin- and DHM-treated mice, the plaque numbers and IR were all not significant changed as compared to their respective controls [Plaque number: experiment Ⅰ, control Ⅰ=163.5 (144.8–185.8), baicalin=154.0 (122.8–165.8), *P*>0.05, ***Fig. 7A***; experiment Ⅱ, control Ⅱ=149.0 (128.5–197.8), DHM=136.0 (117.5–165.8), *P*>0.05, ***Fig. 7C***. IR area: experiment I, control Ⅰ=0.82% (0.77%–0.96%), baicalin=0.63% (0.47%–0.89%), *P*>0.05, ***Fig. 7E***; experiment Ⅱ, control Ⅱ=0.67% (0.56%–0.77%), DHM=0.58% (0.50–0.74), *P*≤0.05]. From hippocampus, similar data of plaque numbers and IR areas were recorded [plaque number: experiment Ⅰ, control Ⅰ=21.0 (16.5–31.8), baicalin=19.0 (14.3–26.3), *P*>0.05, ***Fig. 7A***; experiment Ⅱ, control Ⅱ=21.5 (16.8–29.8), DHM=15.5 (9.5–21.3), *P*>0.05, ***Fig. 7C***. IR area: experiment Ⅰ, control Ⅰ=0.69% (0.56%–0.87%), baicalin=0.57% (0.45%–0.79%), *P*>0.05, ***Fig. 7C***; experiment Ⅱ, control Ⅱ=0.48% (0.44%–0.85%), DHM=0.42% (0.39%–0.64%), *P*>0.05, ***Fig. 7E***]. Our previous results^[16]^ showed a significant reduction in plaque numbers in the cortex of icariin- and hesperidin-treated mice [experiment Ⅱ, control Ⅱ=149.0 (128.5–197.8), icariin=100.5 (61.8–130.3), *P*≤0.05, ***Fig. 7C***; experiment Ⅲ, control Ⅲ=157.0 (147.0–169.3), hesperidin=126.0 (103.0–142.0), *P*≤0.05, ***Fig. 7E***]. In the hippocampus, the difference in the plaque numbers between icariin group and control group was statistically significant [experiment Ⅱ, control Ⅱ=21.5 (16.8–29.8), icariin=11.5 (4.8–15.5), *P*≤0.05, ***Fig. 7C***]. After ten days of icariin and hesperidin administration, the Aβ IR areas were significantly reduced in the cortex [experiment Ⅱ, control Ⅱ=0.67% (0.56%–0.77%), icariin=0.16% (0.11%–0.38%), *P*≤0.05, ***Fig. 7D***; experiment Ⅲ, control Ⅲ=0.72% (0.58%–0.87%), hesperidin=0.41% (0.34%–0.47%), *P*≤0.05, ***Fig. 7F***]; in the hippocampus of hesperidin-treated mice, the Aβ IR areas were also significantly reduced [experiment Ⅲ, control Ⅲ=0.48% (0.34%–0.70%), hesperidin=0.24% (0.16%–0.41%), *P*≤0.05, ***Fig. 7F***].

### Remediation of affiliative behavior impairment of APP/PS1 mice (nest construction assay)

Nest building is one type of affiliative, social behavior which is of great importance to the survival of an animal. Our previous studies showed that the nesting ability of the transgenic mice was impaired in comparison to normal mice^[10]^, which might be the result of anxiety (neophobia), hypolocomotion, and/or a reduction in normal chewing tendencies^[17]^.

**Fig.8:**
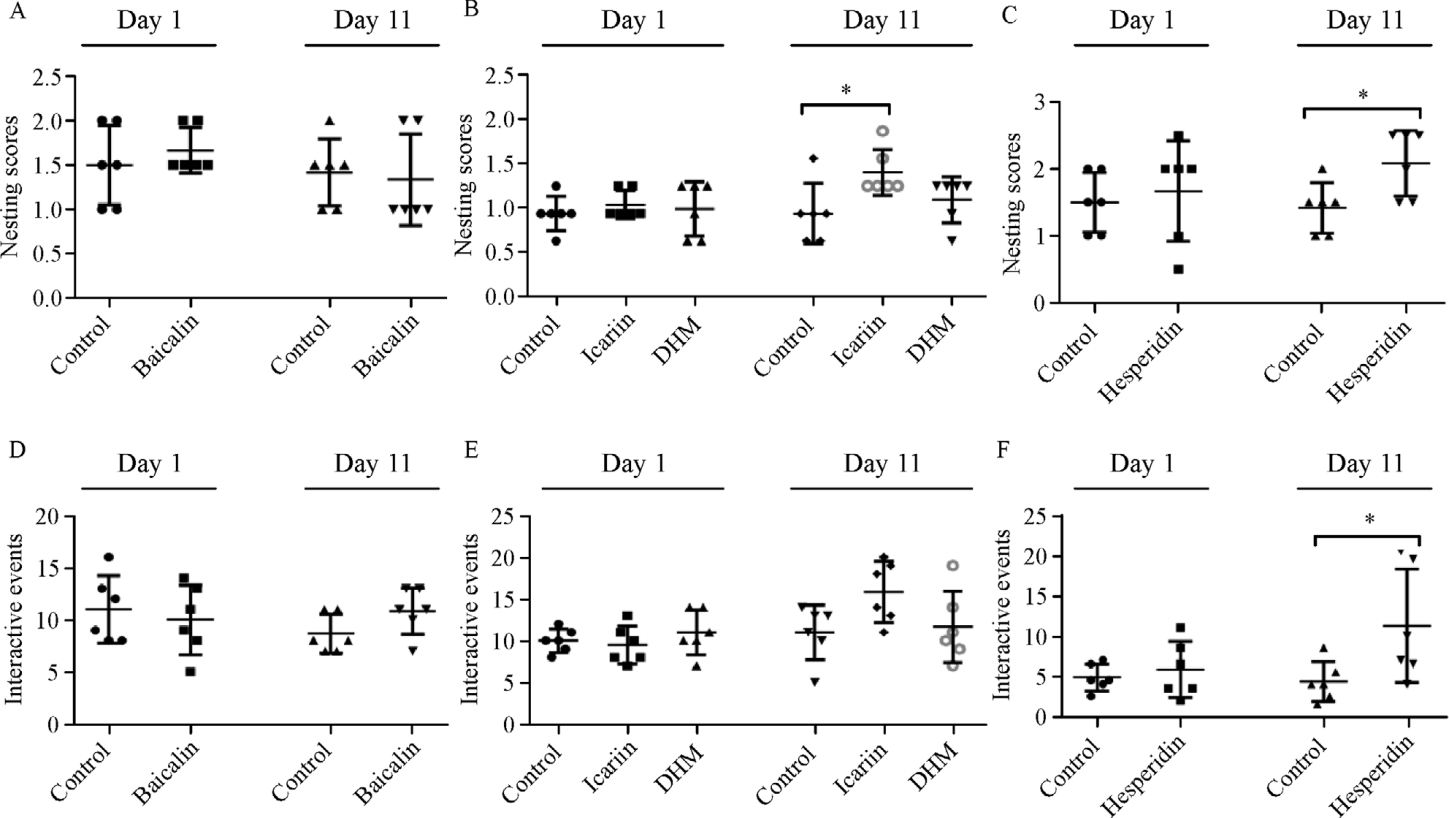
**Effect of four polyphenols on impaired nesting ability and resident-intruder assay. **

Prior to treatment, nest building performance of all groups was not significantly different (***Fig. 8A***, ***B ***and*** C***). After ten days of treatment (day 11), the nesting scores of baicalin- and DHM-treated remained unchanged [experiment Ⅰ, control Ⅰ=1.50 (1.00–1.63), baicalin=1.00 (1.00–2.00), *P*>0.05, ***Fig. 8A***; experiment Ⅱ, control Ⅱ=1.50 (1.00–1.75), DHM=2.00 (1.38–2.00), *P*>0.05, ***Fig. 8B***], while nests built by icariin- and hesperidin-treated mice were of improved quality as indicated by significant differences in nesting scores [experiment Ⅱ, control Ⅱ=1.50 (1.00–1.75), icariin=2.00 (2.00–2.63), *P*≤0.05, ***Fig. 8B***; experiment Ⅲ, control Ⅲ=1.50 (1.00–1.63), hesperidin=2.25 (1.50–2.50), *P*≤0.05, ***Fig. 8C***]^[16^,^18]^. Moreover, the baicalin- and DHM-treated control transgenic mice investigated and slightly chewed on paper towels.They did not really destruct the paper towels like they did just ten days ago, however paper towels were found all over the cage grouped and non-grouped away from any corners. In sharp contrast, relatively immediate chewing and tearing of the paper towels was observed in icariin- and hesperidin-treated mice; the paper towels were torn into pieces and grouped into a corner.

### Remediation of social interaction impairment of APP/PS1 mice (resident-intruder assay)

In the social interaction assay, no matter before or after treatment, the distances traveled were not statistically significant between treatment and their respective control groups (data not shown)^[18]^.

Two unfamiliar mice placed in the same cage will often display high levels of sniffing, following, grooming, rearing at the other mouse, sitting or lying next to the other mouse and so on. The resident-intruder sessions were videotaped and defined behavioral events were counted. Prior to treatment, the difference ininteractive behavior counts between groups was not statistically significant (***Fig. 8 D***, ***E ***and*** F***). Following ten days of treatment, interactive behavior events in baicalin-, icariin- and DHM-treated mice were not significantly different compared to vehicle-treated mice [experiment Ⅰ, control Ⅰ=8.00 (7.00–11.00), baicalin=11.00 (9.25–14.00), *P*>0.05, ***Fig. 8D***; experiment Ⅱ, control Ⅱ=12.00 (8.75–13.25), icariin=16.00 (12.50–19.25), DHM=10.50 (8.50–15.25), *P*>0.05, ***Fig. 8E***]. Hesperidin-treated arm showed a significant higher frequency of interactive behaviors as compared to control mice [experiment Ⅲ, control Ⅲ=8.00 (4.50–12.50), hesperidin=17.00 (12.00–39.50), *P*≤0.05, ***Fig. 8F***]^[18]^.

Because of compensation, the frequencies of independent behaviors in all polyphenol-treated mice were higher compared to control mice. However, the differences were not statistically significant (data not shown). At the same time, there was no difference in control groups between day one and day eleven (data not shown).

## Discussion

Epidemiological evidences suggest that regular consumption of vegetables and fruits with a high polyphenol content could reduce the incidence of some age-associated neurological diseases^[19]^. These findings are supported by *in vivo* models of neurological disorders, revealing that polyphenol-rich plant extracts have neuroprotective effects and can even reversed cognitive deficits^[20]^. Notably, in prevention and treatment of neurodegeneration, polyphenols have gathered much interest and effects at different sites within the known AD pathways.

Virtual drug screening is a powerful approach to gain an overview of complex drug-protein interaction^[21]^. We have used this approach to study polyphenol binding to proteins of the “Alzheimer’s disease pathway” from Kyoto Encyclopedia of Genes and Genomes (KEGG) pathway database. Surprisingly, baicalin, DHM, icariin and hesperidin were docked to several proteins of this pathway with a very similar pattern of potential target proteins. Some of these targets were anticipated, like binding to β-amyloid. APP is a long protein formed by up to 771 amino acids. It is produced in large quantities in neurons and is metabolized very rapidly. According to the “amyloid hypothesis”, Aβ peptides are produced through the endoproteolysis of APP^[22]^. The enzymes that cleave APP have been extensively characterized. BACE1, a transmembrane aspartic protease, makes the first cut in APP, and produces the secreted sAPP β-ectodomain and the membrane-bound C-terminal fragment C99. Neurons from BACE1^−/−^mice do not produce Aβ, which confirms that BACE1 is the principal β-secretase in neurons and suggests focusing on the design of therapeutics to inhibit BACE1 activity^[23]^. γ-secretase is a multicomponent protease consisting of four integral membrane proteins, with presenilin-1 (PS1) or presenilin-2 (PS2) as the catalytic components^[24]^. C99 is cleaved by the γ-secretase complex to release Aβ_40_ and Aβ_42_. γ-secretase inhibitors (GSIs), such as CHF5074, strongly inhibit Aβ production by modulating the preferred site of γ-secretase cleavage^[25]^. ADAM10, the physiologically relevant, constitutive α-secretase, cuts APP within the Aβ domain, thus preventing Aβ formation. ADAM10 activation by SIRT1 in a mouse model of AD significantly attenuated Aβ deposition and cognitive deficits^[26]^. Aβ in turn can be degraded by proteases such as insulin degrading enzyme (IDE) and neprilysin (NEP), which are remarkably enhanced by ApoE. The transgenic overexpression of IDE or NEP in neurons significantly reduces Aβ levels and plaque associated with AD pathology^[27]^. Caspase-8 is involved in presenilin/γ-secretase activation and Aβ production in apoptosis. Blocking of caspase activity with caspase-8 inhibitor z-IETD-fmk reduced Aβ production in H4 cell line^[28]^. APP-binding proteins are also involved in Aβ production and brain development: APP-BP1 may inhibit Aβ production by interacting with PS1 under physiological conditions and overexpression of APP-BP1 in neurons causing apoptosis^[29]^; the Fe65 proteins transmit an APP-dependent signal important for neuronal positioning in the developing cortex and overexpression of Fe65 induces a dramatic increase in Aβ secretion^[30]^; not only is GAPD a key enzyme in cellular energy production, but it also plays an important role in neuronal apoptosis^[31]^. In addition, during the progression of AD pathology, Aβ accumulation initiates a chronic inflammatory response in the cerebral cortex which is considered to gradually exacerbate the disease^[32]^.

Neuroinflammation with activation of microglial cells is a much debated issue in AD research, but the KEGG pathway is only listing IL-1β and TNF-α. Thus, we limited our approach to the cytokines and, very unexpectedly, observed docking of all four polyphenols to TNF-α. TNF-αthought to play a crucial role in the self-propagation of neuroinflammation, brain development and normal behaviors^[33]^. Non-steroidal anti-inflammatory drugs (NSAIDs), such as R-Flurbiprofen, PMX205, and CNI-1493, have been claimed to affect the inflammatory process by reducing TNF-α levels and to be beneficial in the treatment of AD^[34]^. Thus, this interesting issue certainly warrants further investigation.

The proteins described above are all potential targets of these four polyphenols (***Fig. 1***–***5***). It had been suggested that baicalin, DHM, icariin and hesperidin might produce anti-AD effects by modulating amyloidosis. Interestingly, the biggest difference among these four binding patterns is that calpain protein being the potential target of DHM and baicalin rather than icariin and hesperidin. It is tempting to speculate that this might be one reason for the observed different *in vivo* effects. Calpain activation promotes BACE1 expression, APP expression and amyloid plaque formation^[35]^. Several calpain inhibitions, such as E64, improved synaptic and memory deficits produced by Aβ in APP/PS1 mice^[36]^. Thus, calpain is a clinically relevant target and potential differential binding of polyphenols merits further investigation.

Although baicalin and DHM were reported to exert neuroprotective effects in several other *in vivo* and *in vitro* studies, they could improve neither the behavioral dysfunction nor pathology. In addition to considering results from our docking experiments to explain differences between disease reducing icariin and hesperidin and non-effective baicalin and DHM, it should be considered that the *in vivo* situation is complex. For example, although baicalin can cross the BBB, the permeability is not high. For instance, after injection of a dose of 24 mg/kg, the concentration of baicalin in cerebrospinal fluid (CSF) was merely 27% of that in serum^[37]^. In contrast, Youdim *et al*.’s study reported that the apparent permeability to cross the *in vitro* BBB model was higher for the citrus flavonoids, especially hesperitin and their more polar glucuronidated conjugates^[38]^. In addition, after oral administration of icariin at the dose of 100 mg/kg, the absolute availability was 12.02% in rats^[39]^; while this parameter was much lower (4.84%) in rats after giving 100 mg/kg of baicalin by gavage^[40]^. Moreover, the excreted amount of icariin from urine, faeces and bile was very small, and the accumulated amount for 24 hours was merely 1.99%, 12.83% and 0.066% of the oral administration dosage (100 mg/kg)^[39]^; in contrast, the excreted amount of DHM was a little higher as about 29.0% of the dose (100 mg/kg) was eliminated *via* faeces^[41]^. Thus, other factors will certainly affect the therapeutic effects of some polyphenols in animal models of AD.

In summary, molecular docking revealed a potential interaction of polyphenols with several proteins of the AD pathway. Some of the proteins were known targets of polyphenols, others are potential novel targets. Thus, the docking procedure revealed many very interesting and previously unknown targets. It further shows that it might be difficult to pin-down polyphenol effects interacting with only a single protein, but maybe the promiscuous binding to several proteins will result into a complex net effect.

*In vivo* data supports the anti-amyloidosis effects of some polyphenols. Considering the patterns of *in silico* binding, the only discriminator between effective and non-effective polyphenols is the binding to calpain. While this is a noteworthy result, it should be considered that additional pharmacological factors will certainly contribute to *in vivo* effects.
